# Expression of epigenetic pathway related genes in association with PD-L1, ER/PgR and MLH1 in endometrial carcinoma

**DOI:** 10.1371/journal.pone.0264014

**Published:** 2022-02-28

**Authors:** Ozlen Saglam, Biwei Cao, Xuefeng Wang, Gokce A. Toruner, Jose R. Conejo-Garcia

**Affiliations:** 1 Department of Pathology, Moffitt Cancer Center, Tampa, FL, United States of America; 2 Department of Biostatistics and Bioinformatics, Moffitt Cancer Center, Tampa, FL, United States of America; 3 Department of Hematopathology, University of Texas MD Anderson Cancer Center, Houston, TX, United States of America; 4 Department of Immunology, Moffitt Cancer Center, Tampa, FL, United States of America; University of East Anglia, UNITED KINGDOM

## Abstract

The distribution of Endometrial Cancer (EC)-related deaths is uneven among the morphologic subtypes of EC. Serous Cancer (SC) makes 10% of all EC and accounts for 40% of EC-related deaths. We investigated expression of selected genes involved in epigenetic pathways by immunohistochemistry in a cohort of 106 EC patients and analyzed mRNA-based expression levels for the same set of genes in EC samples from The Cancer Genome Atlas (TCGA) dataset. A tissue microarray was constructed using low-grade (n = 30) and high-grade (n = 28) endometrioid, serous (n = 31) and clear cell carcinoma (n = 17) samples. Epigenetic marker levels were associated with PD-L1, ER/PgR, and MLH1 expression. Epigenetic markers were evaluated by H-score and PD-L1 expression was recorded by using Combined Positive Score. Results were correlated with disease stage and survival outcome. BRD4, KAT6a and HDAC9 levels were higher in SC compared to other histologic subtypes (p<0.001–0.038). After adjusting for multiple comparisons, DNMT3b expression was higher in SC compared to endometrioid-type but not between SC and CCC. The expression levels of BRD4 (p = 0.021) and KAT6a (p = 0.0027) were positively associated with PD-L abundance, while PgR (p = 0.029) and PD-L1 expression were negatively associated. In addition, BRD4 expression was low in specimens with loss of MLH1 expression (p = 0.02). More importantly, BRD4 abundance had a negative impact on disease outcome (p = 0.02). Transcriptionally, *BRD4*, *KAT6a* and *DNMT3b* expression levels were higher in SC in TCGA dataset. The median *PD-L1* expression was marginally associated with *BRD4*, a transcriptional activator of *CD274/PD-L1* (p = 0.069) and positively with *KAT6a* (p = 0.0095). In conclusion, the protein expression levels of epigenetic markers involved in cancer pathogenesis are increased by immunohistochemistry in SC. *PD-L1* levels are associated with *BRD4* and *KAT6a* in EC samples. A combination therapy with *BRD4/PD-L1* or *KAT6a/PD-L1* inhibitors might have a potential use in EC, in particular serous-type carcinoma.

## Introduction

Endometrial cancer (EC) makes more than 90% of uterine corpus malignancies in the United States. In 2020 there were 65,620 new uterine cancer diagnoses with the estimated death rates of 12,590 [1] Unlike most other cancer types, the incidence of EC and associated mortality rates are still increasing. Among the histologic subtypes of EC, there is an uneven distribution of cancer-related deaths [2]. For instance, serous cancers (SC) represent approximately 10% of all EC but account for 40% of EC-related deaths [3, 4]. Surgery is the mainstay of the initial management of all EC. Patients with stage I SC have an increased risk of extrapelvic recurrence, and adjuvant therapy including systemic chemotherapy and vaginal brachytherapy is generally recommended without definite survival benefits [2]. The management of advanced and recurrent EC is also challenging. Therefore, molecular features might dictate disease management and clinical outcome [5].

The somatic copy number alterations are more frequently observed in the pathogenesis of SC compared to other morphologic subtypes. SC overlaps with the “copy number (CN) high” group at the molecular level to such an extent; in the current molecular classification of EC the CN-high group is also known as “serous-like” carcinoma [6]. In our prior analysis of EC samples from The Cancer Genome Atlas (TCGA) dataset, a group of genes involved in the epigenetic pathways are found to be amplified in SC [7]. These include Bromodomain-containing protein 4 (*BRD4*) and the lysine acetyltransferase 6 (*KAT6a*). Epigenetic, immunologic and hormonal pathways, and DNA repair-related genes have complex interactions with each other at multiple levels. Both *BRD4* and *KAT6a* have a potential role in hormone dependent cancers. *BRD4* activity is required for proliferation of Estrogen Receptor (ER)-positive breast and endometrial cancer cell lines [8] *KAT6a* activates ER-alpha expression in breast cancer [9].

*BRD4* inhibition is also known to promote anti-tumor immunity by suppressing Programmed death-ligand 1 (*PD-L1*) expression [10]. MutL Homolog 1 (*MLH1)*, a DNA repair gene, and Progesterone Receptor (*PgR*) are frequently epigenetically silenced in EC. Histone deacetylase (*HDAC*) inhibitors are able to restore the expression of *PgR* and might facilitate hormonal therapy [11]. Among HDAC family members, *HDAC9* deficiency particularly promoted tumor progression by decreasing peritumoral inflammation in animal models [12]. *HDAC9* decreased ER-alpha mRNA and protein expression, and inhibited its transcriptional activity in breast cancer cell lines [13]. Similarly, elevated expression of DNA methyltransferase 3b (*DNMT3b*) was significantly associated with absence of ER-alpha and higher histologic grade [14]. The latter findings suggest potential involvement of *DNMT3b* with aggressive behavior of breast cancer. DNA methyltransferase family enzyme levels are involved in EC pathogenesis by methylation of *PgR* [15] Reportedly, *DNMT3b* expression levels varied among morphologic subtypes and histologic grades of EC [16, 17].

In order to explore these molecular interactions further in EC, we constructed a Tissue Microarray (TMA) using clinical samples from 106 patients with EC of multiple histologic types. The protein expression levels of *BRD4*, *KAT6a*, *DNMT3b and HDAC9* by immunohistochemistry were associated with *PD-L1*, *MLH1*, *ER*, and *PgR* levels in histologic subtypes of EC. The marker expression was analyzed against clinicopathologic parameters. Finally, we used the EC dataset of TCGA to validate our results.

## Material and methods

### TMA and immunohistochemistry

Moffitt Cancer Center institutional review board approval (Advarra IRB #19196) was followed by construction of a TMA. The Scientific Review Committee waived the requirement for informed consent because only archival material was used (retrospective study). All data were fully anonymized. We used formalin-fixed paraffin embedded primary EC samples. Duplicate 1.0 mm cores were sampled to account for tissue heterogeneity. In total there were 106 EC samples with distribution of 30 FIGO grade 1–2 (low-grade) endometrioid (LEMC) (28.3%), 28 FIGO grade 3 (high-grade) HEMC (26.4%), 31 SC (29.2%), and 17 Clear Cell Carcinoma (CCC) (16%). All pathology slides were reviewed by two gynecologic pathologists, and primary diagnosis and histologic grade were confirmed. The patient’s age at diagnosis, disease stage, presence/absence of lymphovascular invasion (LVI), survival outcomes were recorded for each sample.

Slides were stained using a Ventana Discovery XT automated system (Ventana Medical Systems, Tucson, AZ) as per manufacturer’s protocol with proprietary reagents for PD-L1*(13684*, *Cell Signaling Technologies*, *Danvers*, *MA)* ER *(790–4324*, *Ventana)*, PgR *(790–2223*, *Ventana)*, MLH1 (#790–5091, Ventana) and DNMT3b (ab227883, Cambridge, MA) antibodies. Leica Bond RX automated system (Leica Biosystems, Buffalo Grove, IL) was used for BRD4 (ab128874, Abcam, Cambridge, MA), KAT6a (PA5-66566, Invitrogen, Carlsbad, CA), and HDAC9 (MA5-26729, Invitrogen). After deparaffinization, heat induced antigen retrieval was performed for the followings: PD-L1, DNMT3b, ER/PgR, MLH1, and KAT6a. Epitope Retrieval Solution was used for 20 and 15 minutes for BRD4 and HDAC9 respectively. At the final stage, slides were dehydrated and coverslipped. S1 Appendix was submitted for detailed descriptions of immunohistochemical protocols.

The expression of epigenetic markers was evaluated by using H-score (range: 0–300). H-score was calculated by multiplying the percentage of positively stained cells by nuclear staining intensity. The scoring of immunostains was blinded. Readings from multiple cores were averaged. PD-L1 expression was recorded by using Combined Positive Score (CPS). The number of positively stained tumor cells, lymphocytes, and macrophages was divided by the total number of viable tumor cells and multiplied by 100. Any sample with CPS of 1 or higher membranous positivity was considered a “PD-L1-expressing” tumor. The cut-off was 1% nuclear positivity for MLH1 expression.

### TCGA and statistical analysis

Gene expression (mRNA) and clinical data of EC samples from TCGA dataset were downloaded from the cBio web portal (PMID: 23550210) by selecting “Uterine Corpus EC (TCGA, PanCancer Atlas)” for validation. The downloaded normalized gene expression values (RSEM) were log2-transformed before statistical analyses. Kruskal-Wallis test was used to compare the gene expression levels in tumor subtypes. Holm-Bonferroni method was used for correction of multiple comparisons. Wilcoxon signed-rank test was performed to test the association between epigenetic marker expressions and *PD-L1*. After stratification of patients based on median gene expression, Kaplan-Meier curves, log-rank test and Cox proportional-hazard regression model were further employed to investigate the association between gene expression values and overall survival (OS). The survival outcomes of patients with tumors expressing higher than median Interferon gamma (*IFNG*) and *PD-L1* mRNA levels were compared to survival of patients with tumors expressing higher *BRD4/PD-L1* levels. All statistical analyses were performed using R version 4.0.2. The gene expression values were visualized by boxplots using R package ggpubr. Clinical and TCGA samples were analyzed by the same methodologies.

## Results

### Clinical samples

The median age at diagnosis was 64.5 years for the entire clinical cohort. The median age at diagnosis for EMC, SC, and CCC was 61, 70 and 64 years respectively. The staging procedure was performed on 94 cancers. There were 59 early-stage (FIGO stage I and II) (63%), and 35 late-stage (FIGO stage III and IV) diseases (37%). Patients diagnosed at an earlier age had significantly better OS compared to patients diagnosed at later ages in Multivariable Cox Proportional Hazard Model: 1.078 [95% CI 1.035–1.123] (p = 0.0003). The disease stage was associated with disease outcome (p = 0.0001). LVI was identified in 45 cancers (43%). Presence of LVI was associated with adverse disease outcome with hazard ratio of 3.769 [95% CI 1.446, 9.824] (p = 0.0066). The summary of clinical data was presented in S1 Table.

### SC had the highest BRD4, KAT6a and HDAC9 expression by immunohistochemistry

**Fig 1:**
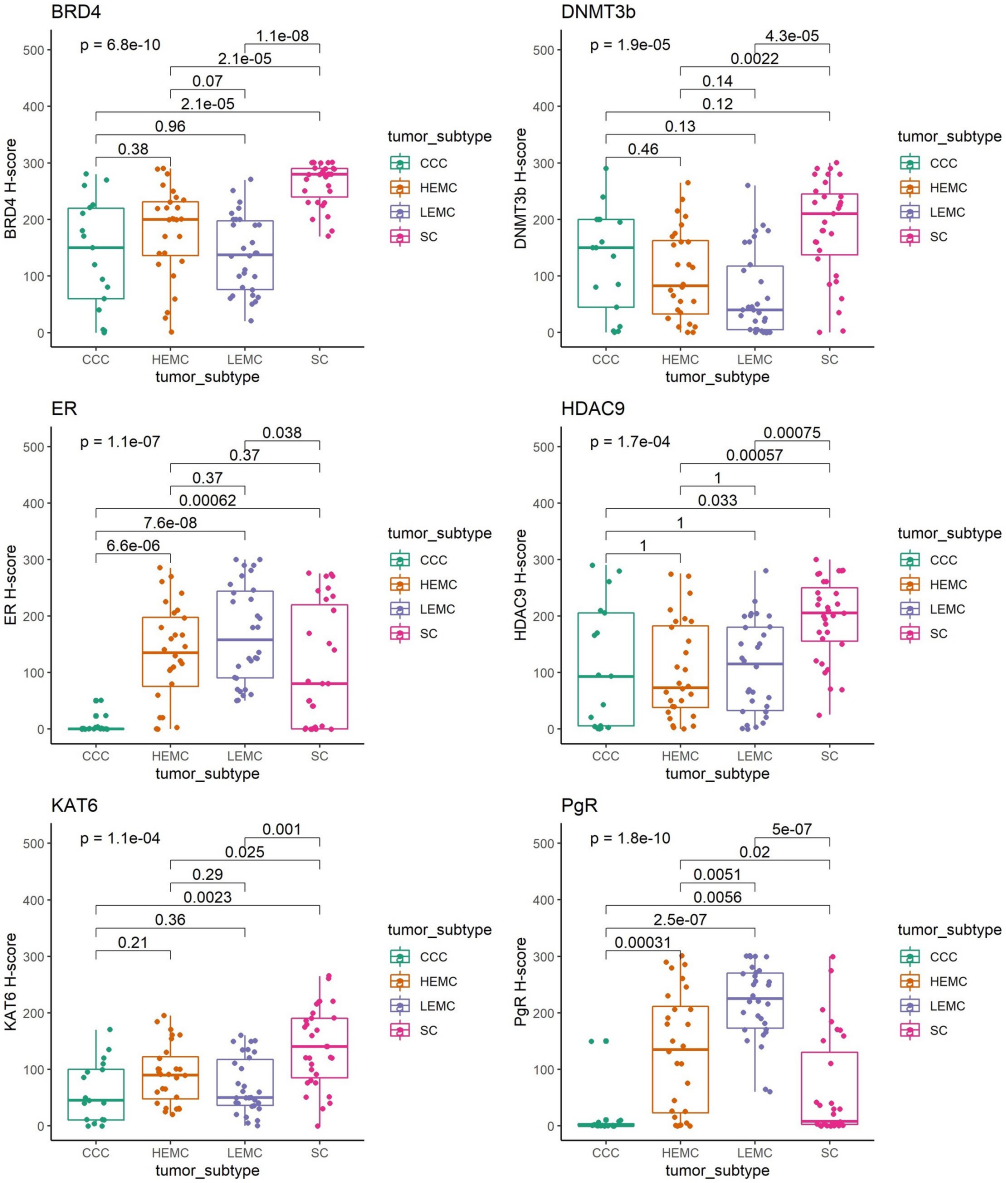
The box plots for marker expression against tumor subtype: Kruskal Wallis test was used to compare the protein expression levels by immunohistochemistry among the morphologic subtypes of endometrial carcinoma. CCC: Clear Cell Carcinoma. HEMC: High-grade Endometrioid Cancer. LEMC: Low-grade Endometrioid Cancer. SC: Serous cancer.

**Fig 2:**
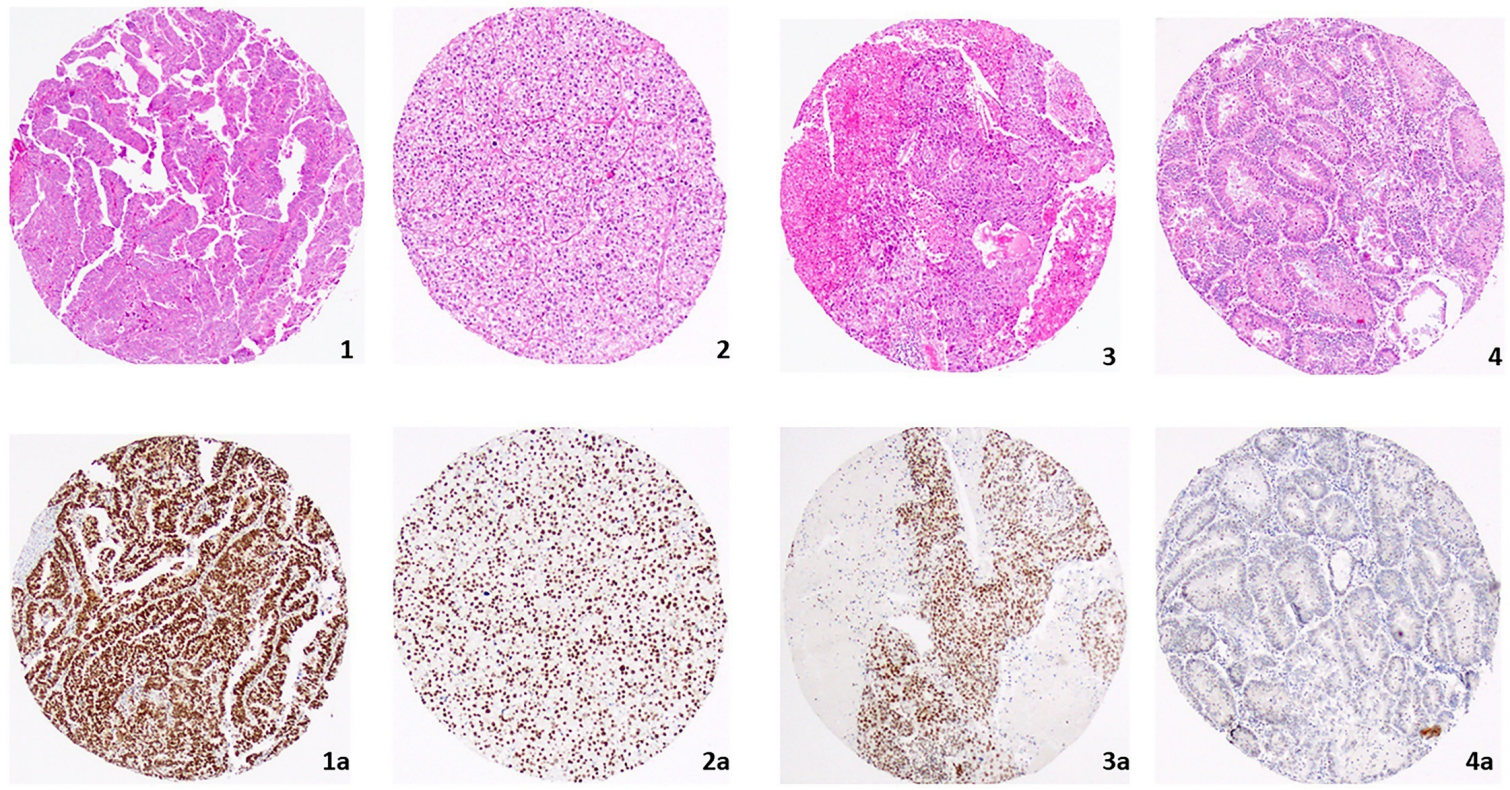
*BRD4* expression by immunohistochemistry in histologic subtypes of endometrial carcinoma: 1: Serous-type endometrial cancer. 1a: H-score: 300. 2: Clear Cell Carcinoma. 2a: H-score: 210. 3: High-grade endometrioid endometrial cancer. 3a: H-score: 240. 4. Low-grade endometrioid endometrial cancer. 4a: H-score: 20.

The median H-score for BRD4, HDAC9, DNMT3b *and* KAT6a were 200, 140, 120 and 90 respectively in all EC subgroups (Table 1). In SC, the median marker expression level was the highest for all epigenetic markers (p<0.001 to 0.038) except DNMT3b (Fig 1). After adjusting for multiple comparisons with Holm’s method, DNMT3b expression was higher in SC compared to HEMC (p = 0.0022), and LEMC (p < 0.0001) but there was no difference in expression levels between SC and CCC (p = 0.12). BRD4 expression levels by immunohistochemistry among the histologic subtypes (SC, CCC, HEMC, LEMC) of EC were illustrated in Fig 2. HEMC had the second highest epigenetic marker expression levels. However, only BRD4 expression level was marginally higher compared to LEMC (p = 0.07) (Fig 1). DNMT3b expression was higher than median in 43% of HEMC. Only eight out of 30 LEMC had an equal or higher median H-scores for DNMT3b (Table 2). LEMC had the lowest median H-score for all markers except HDAC9. The median H-score of HDAC9 was higher in LEMC compared to CCC and HEMC. ER and PgR expression levels were low in CCC and they were expressed at the highest levels in LEMC (p<0.001) (S1 Fig). The HDAC9 (p = 0.012) and BRD4 (p = 0.044) medians were significantly associated with ER.

**Table 1:** Summary of epigenetic marker expression: The median H-score and the range of marker expression (within brackets) are presented for each morphologic subtype.

	BRD4	KAT6a	HADC9	DNMT3b
**Epigenetic Marker expression for all histologic subtypes**	200 [0–300]	90 [0–265]	140 [0–300]	120 [0–300]
**Serous carcinoma**	280 [170–300]	140 [0–265]	205 [25–300]	210 [0–300]
**Low-grade Endometrioid cancer**	137.5 [20–270]	50 [0–160]	115 [0–280]	40 [0–260]
**High-grade Endometrioid cancer**	200 [0–290]	90 [20–195]	72.5 [0–275]	82.5 [0–265]
**Clear cell carcinoma**	150 [0–280]	45 [0–170]	92.5 [0–290]	150 [0–290]

**Table 2:** The comparison of marker expression among the histologic types of endometrial carcinoma: The distribution of cases around the median marker expression is listed.

	CCC	HEMC	LEMC	SC	P overall
	*N = 17*	*N = 28*	*N = 30*	*N = 31*	
**PDL1:**					0.268
E	8 (47.1%)	14 (50.0%)	8 (26.7%)	14 (45.2%)	
NE	9 (52.9%)	14 (50.0%)	22 (73.3%)	17 (54.8%)	
**BRD4:**					<0.001
<median	11 (64.7%)	11 (39.3%)	22 (73.3%)	2 (6.45%)	
> = median	6 (35.3%)	17 (60.7%)	8 (26.7%)	29 (93.5%)	
**DNMT3b:**					0.001
<median	7 (41.2%)	16 (57.1%)	22 (73.3%)	7 (22.6%)	
> = median	10 (58.8%)	12 (42.9%)	8 (26.7%)	24 (77.4%)	
**HDAC9:**					0.003
<median	10 (58.8%)	19 (67.9%)	17 (56.7%)	7 (22.6%)	
> = median	7 (41.2%)	9 (32.1%)	13 (43.3%)	24 (77.4%)	
**KAT6:**					0.006
<median	11 (64.7%)	12 (42.9%)	20 (66.7%)	8 (25.8%)	
> = median	6 (35.3%)	16 (57.1%)	10 (33.3%)	23 (74.2%)	
**ER:**					<0.001
<median	17 (100%)	9 (32.1%)	9 (30.0%)	18 (58.1%)	
> = median	0 (0.00%)	19 (67.9%)	21 (70.0%)	13 (41.9%)	
**PgR:**					<0.001
<median	15 (93.8%)	11 (39.3%)	2 (6.67%)	22 (71.0%)	
> = median	1 (6.25%)	17 (60.7%)	28 (93.3%)	9 (29.0%)	

E: expressed. NE: not expressed. CCC: Clear Cell Carcinoma. HEMC: high-grade endometrioid Cancer. LEMC: Low-grade endometrioid Cancer. SC: serous carcinoma.

### PD-L1 expression was positively associated with H-scores of BRD4 and KAT6a and negatively with PgR

**Fig 3:**
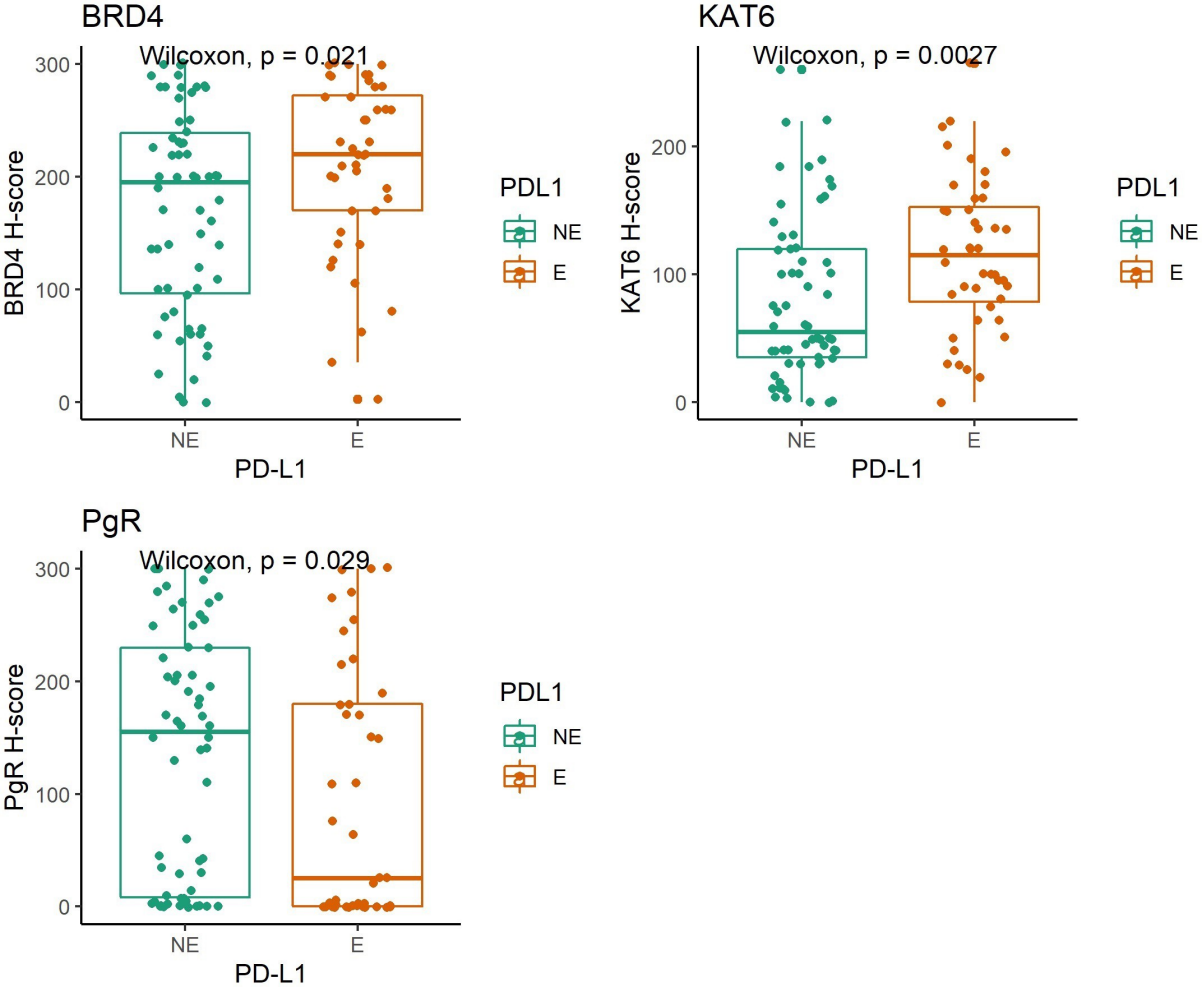
Marker expression against *PD-L1*: Wilcoxon signed-rank test was performed to explore the association between H-scores of epigenetic markers and PD-L1 expression by immunohistochemistry. There is a positive association between PD-L1, BRD4, and KAT6a and negative association between PD-L1 and PgR levels. NE: Not expressed. E: Expressed.

PD-L1 was expressed in 44 samples out of 106. The majority of samples had a CPS of 10 or less (n = 89). CPS was more than 10 in 17 samples (16%). PD-L1 expression levels did not show any significant difference among tumor subtypes. The highest expression levels were observed in HEMC samples (50% of samples) and the lowest expression in LEMC. Only eight out of 30 LEMC expressed PD-L1 (27%). There was a positive association between PD-L1 and H-scores of BRD4 (p = 0.021) and KAT6a (p = 0.0027). The PgR expression levels were inversely associated with PD-L1 expression (p = 0.029) (Fig 3). Other epigenetic markers were not associated with PD-L1 expression. MLH1 expression was intact in the majority of samples (S2 Table). The loss of expression was identified in 23 samples out of 106 (22%). The most common tumor subtype with loss of expression was HEMC (n = 13 samples) followed by LEMC (n = 9), and CCC (n = 1). Low median H-score of BRD4 expression was associated with MLH1 loss expression (p = 0.02) (S2 Fig). There was no association between MLH1 and PD-L1 expression levels (p = 0.16).

### BRD4 protein expression was associated with adverse disease outcome in clinical samples

**Fig 4:**
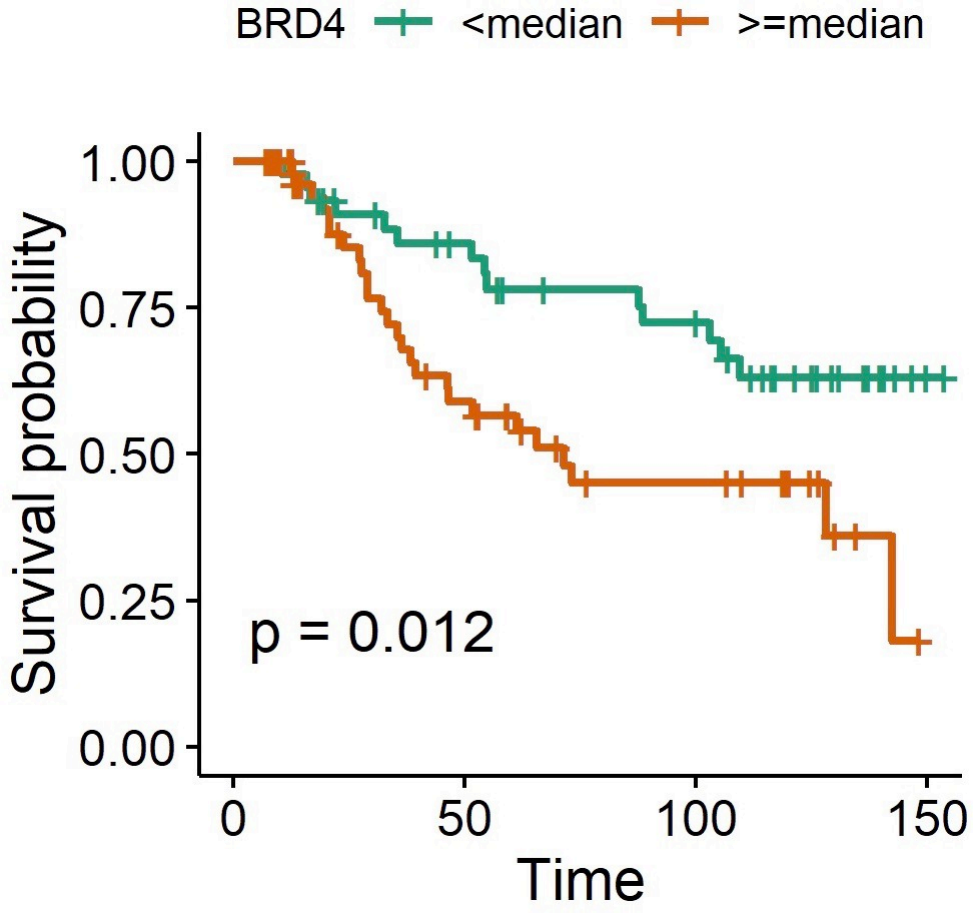
BRD4 expression against overall survival (Kaplan-Meier Curve): In clinical samples, BRD4 protein expression levels were negatively associated with overall survival in multivariable Cox proportional-hazards model.

The median disease survival for late-stage cancers was 48.3 months. The survival rates were not estimable for early-stage diseases due to limited clinical follow-up. The backward elimination was done by adjusting for age at diagnosis, tumor subtype, disease stage and LVI (Table 3). BRD4 expression levels were negatively associated with OS with the HR of 2.9 [95% CI 1.1–7.7] (p = 0.02) (Fig 4). PD-L1 had borderline significance for better survival rates (p = 0.052). Other epigenetic markers did not have any impact on disease outcome.

**Table 3:** Multivariable Cox proportional-hazards regression model for overall survival after backward elimination: The backward elimination was done by adjusting for age at diagnosis, tumor subtype, disease stage and lymphovascular (LVI) invasion.

Variable	Level	HR (95% CI)	P Value
**Diagnosis**	CCC	1.0 (Reference)	
	HEMC	0.323 (0.086, 1.215)	0.0944
	LEMC	0.266 (0.067, 1.051)	0.0589
	SC	0.441 (0.142, 1.370)	0.1568
**stage group**	I/II	1.0 (Reference)	
	III/IV	1.618 (0.574, 4.559)	0.3629
**LVI**	N	1.0 (Reference)	
	Y	3.769 (1.446, 9.824)	0.0066
**Age at Diagnosis**		1.078 (1.035, 1.123)	0.0003
**BRD4**	< Median	1.0 (Reference)	
	≥ Median	2.929 (1.112, 7.711)	0.0296
**PDL1**	NE	1.0 (Reference)	
	E	0.453 (0.204, 1.006)	0.0517

CCC: Clear cell carcinoma. HEMC: high-grade endometrioid cancer. LEMC: Low-grade endometrioid cancer. SC: Serous cancer. HR: Hazard Ratio. LVI: Lymphovascular invasion.

### TCGA analysis

To validate our results using an independent cohort of EC patients, we next analyzed TCGA datasets. This cohort is composed of 108 SC and 397 EMC (n = 505), but does not include other histological subtypes. Among endometrioid-type there were 213 FIGO grade 1 and 2 (40%) and 292 FIGO grade 3 (60%) cancers. *BRD4* and *DNMT3b* mRNA levels were significantly higher in serous-type compared to EMC (p = 5.1e-09 and 8.7e-15 respectively).

**Fig 5:**
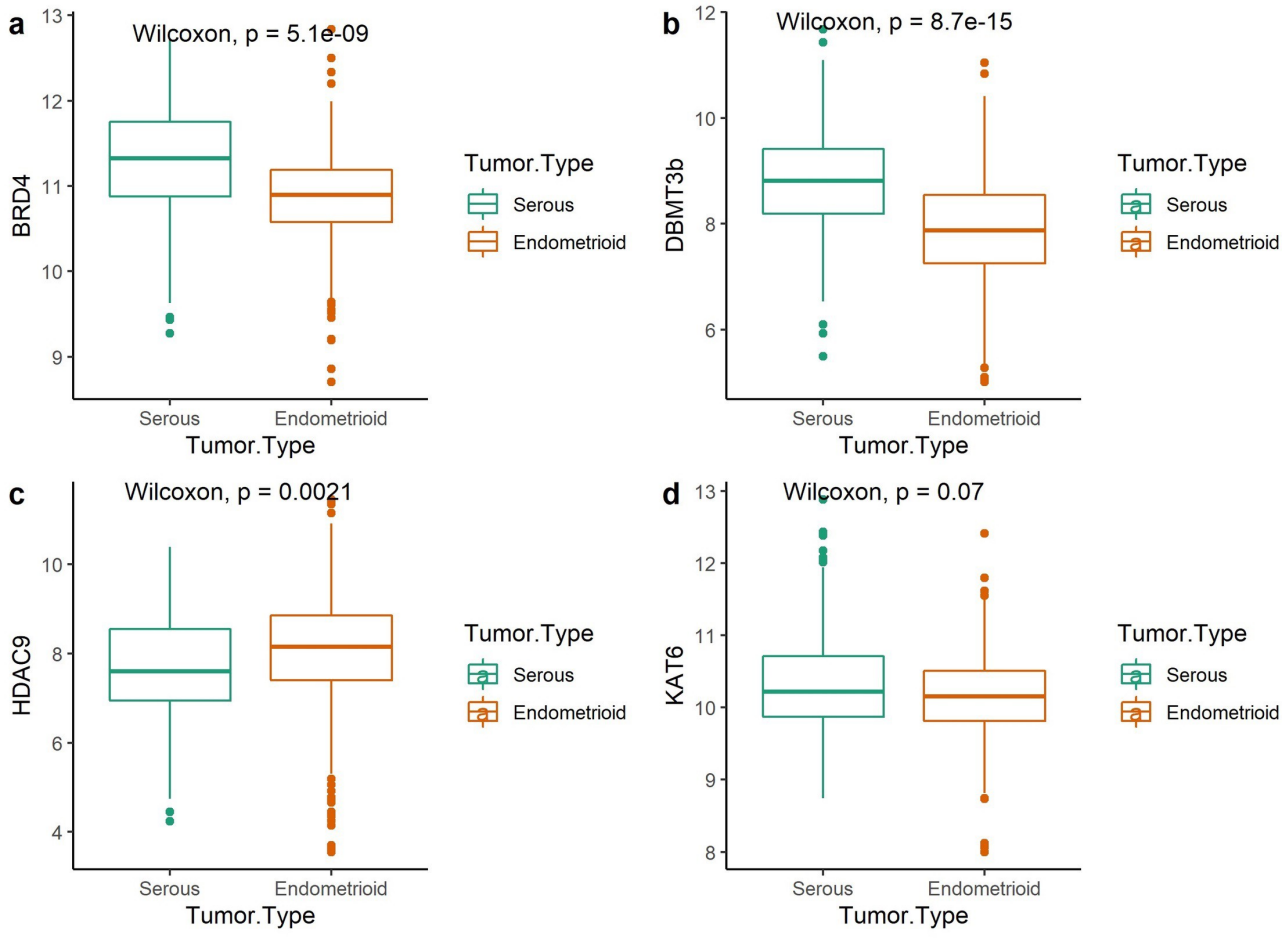
mRNA expression levels of epigenetic markers in EC samples of TCGA dataset; a) *BRD4* b) *DNMT3b* c) *HDAC9* d) *KAT6a* expression levels in serous versus endometrioid cancers. The normalized gene expression values (RSEM) were log2-transformed.

**Fig 6:**
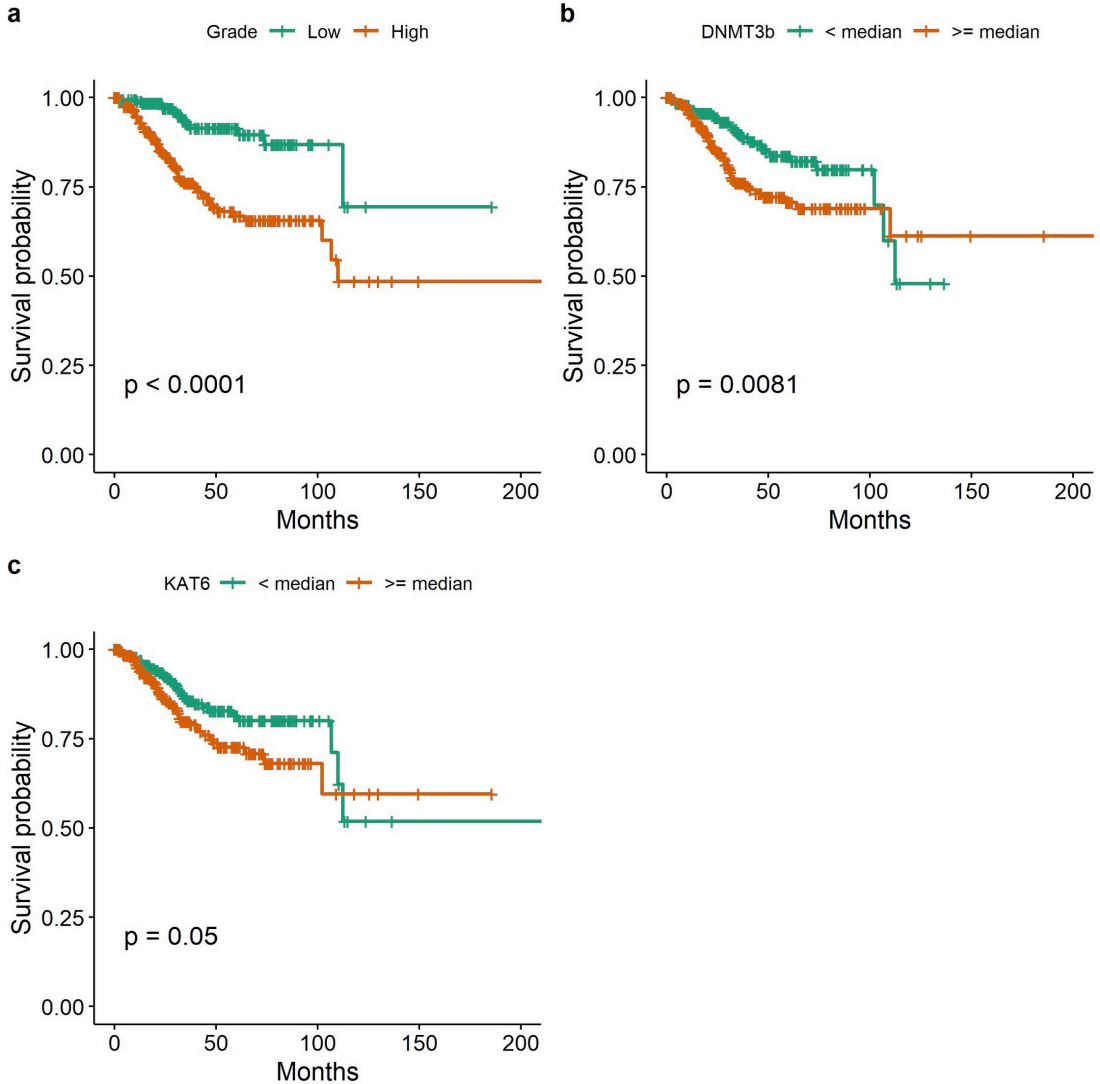
Epigenetic marker expression against survival in TCGA dataset: Kaplan-Meier Curves stratify patients by (a) tumor grade; (b) *DNMT3b* expression level against OS for the entire TCGA cohort; (c) *KAT6a* expression level against OS in SC.

In contrast to results from protein expression in our TMA clinical samples, *HDAC9* was expressed at higher levels in EMC (p = 0.002) not in serous-type and *KAT6a* median expression was only borderline higher in SC (p = 0.070) (Fig 5). *ER* and *PgR* were expressed at higher levels in EMC. *PD-L1* expression was associated with median expression levels of epigenetic markers. The median *PD-L1* expression was marginally associated with the median of *BRD4* (p = 0.069) levels (S3 Fig). The positive association between *KAT6a* expression and median *PD-L1* expression in TCGA data was supportive of results from immunohistochemical analysis in our TMA clinical samples (p = 0.0095). However, there was no association between *PD-L1* and *PgR* in TCGA analysis. We also found that there was a borderline correlation between *BRD4* and *KAT6a* expression levels (r = 0.48) (S4 Fig). The median survival rates for SC, low and high-grade EMC were 27.4, 32.6 and 30.4 months respectively. Patients with LEMC had better OS (p<0.0001). In univariate analysis, the low expression levels of *DNMT3b* were associated with better survival outcomes for the entire cohort (p = 0.0081) (Fig 6). *KAT6a* was marginally associated with survival outcomes (p = 0.05). mRNA levels of *BRD4* were not associated with worse OS in contrast to results from the analyses of TMA clinical samples. Patients with high *IFNG/PD-L1-*expressing tumors did not have any survival difference compared to patients with either high-*BRD4/PD-L1* or low PD-L1 expressing tumors.

## Discussion

Epigenetic modifications have a significant role in cancer biology and the interaction between epigenetic and immunologic pathways is emerging from recent studies. Among the epigenetic markers, BRD4 is one of the most studied Bromo-and-Extra-Terminal (BET) family proteins. *BRD4*-gene rearrangements and mutations have been documented in a number of human cancers [18]. *BRD4* regulates the expression of multiple inflammatory cytokines. The therapeutic targeting of *BRD4* inhibits *BRD4* binding to *c-MYC* promoter and prevents the expression of MYC-dependent target genes in cancer and inflammatory cells [19–21]. In both TMA clinical EC samples and TCGA dataset, *BRD4* was expressed at significantly higher levels in SC compared to other histologic subtypes in our analyses.

The median BRD4 expression levels in TMA clinical and TCGA clinical samples levels were also either positively or marginally positively associated with PD-L1 supporting previously reported BRD4/PD-L1 interaction. *BRD4* is known to be a critical regulator of *PD-L1* expression in tumor cells, dendritic cells and macrophages by directly binding the *CD274* (encoding *PD-L1*) gene promoter, at least in ovarian carcinoma cell-lines [10]. In return, *PD-L1* activates *BRD4* transcription. BET inhibitors down-regulate *PD-L1* expression in tumor and inflammatory cells [22]. Overall, our results demonstrate a positive association between *BRD4* and *PD-L1* in EC samples. This finding suggests a potential treatment option for the highest BRD4 expressing EC histologic subtype, SC. In animal models, targeted inhibition of PD-1/PD-L1 axis by combining anti-PD-1 antibodies and BET inhibitors has synergistic response in MYC-driven lymphomas [22]. *BRD4* inhibitors might have a role in treatment of SC in combination with immune checkpoint inhibitors or be used as a single agent against resistance to *PD-L1* treatment. In addition, the positive association between BRD4 and ER protein levels supports results from EC cell lines [8]. HEMC had marginally higher BRD4 expression compared to LEMC in our TMA clinical samples. BRD4 blockers might also have a role in treatment of estrogen-dependent HEMC.

After stage and histologic grade were adjusted, *BRD4* protein levels had an adverse impact on disease outcome of EC in multivariable analysis and the expression of BRD4 was lower in TMA clinical samples with loss of *MLH1* expression. The latter finding is in agreement with current molecular classification of the EC. The gene amplifications are more common in serous-like (CN-high) carcinoma compared to Mismatch Repair deficient EC. Cancers with mismatch repair deficiency are associated with better response rates to immune checkpoint inhibitors in general [23]. Our results might be complementary with each other in evaluation of potential response to immunotherapy and survival outcomes. However, we could not show a survival difference for mRNA expression of *BRD4* in EC samples of TCGA dataset. When the survival difference was further investigated among patients with tumors expressing either high *IFN*G/*PD*-*L1* or high *BRD4/*PD-*L1* against patients with low PD-L1 expressing tumors no difference was observed. This result indicates that neither high secretion of interferon-gamma nor *BRD4*-driven high *PD-L1* expression has a significant impact on OS in EC, *KAT6a* acetylates histones and nonhistone substrates and is involved in cell-cycle regulation and stem cell maintenance. *KAT6a* frequently mutated in leukemia and solid tumors [24]. *KAT6a* and *TP53* interaction increases the KAT-mediated acetylation of *TP53*. This results in increased activity of *TP53* to drive *p21* expression [25]. *TP53* activation mutation rates are high in serous-type EC [26]. *TP53* activation mutations, but not wild-*TP53* or null-type mutations increase the activity of *KAT6a* [27]. We previously demonstrated *KAT6a* gene amplification in SC samples of TCGA dataset [7]. *KAT6a* amplification was associated with both disease progression and disease-free survival. In current analysis, *KAT6a* overexpression is associated with adverse OS at borderline significance in TCGA dataset when patients are stratified based on median gene expression into equal-size groups. *KAT6a*-amplified samples constitute 20% and nonKAT6a-amplified samples constitute 80% of samples. A positive signal might be diluted by group stratification of EC subtypes. In clinical samples, KAT6a protein levels by immunohistochemistry were overexpressed in SC and associated with PD-L1 expression. TCGA dataset validated the association between *KAT6a* and *PD-L1*. The mechanism *of KAT6a* and *PD-L1* interaction at molecular level is not known to our best knowledge. Finally, in TMA clinical samples *PD-L1* expression was inversely associated with *PgR*. In concordance with this finding, *PD-L1* expression is reportedly the highest in *ER/PgR* negative breast tumors [28]. Inhibiting *KAT6a* with *PD-L1* attenuates metastasis of triple negative breast cancer, and improves survival in animal models [29].

HDACs are involved primarily in the repression of gene transcription by removing charge-neutralizing acetyl groups from the histone lysine tails that results in a more compact chromatin structure [30]. *HDAC9* belongs to class II HDACs, primarily localized in cytoplasm and it is occasionally transferred to the nucleus [31]. In our TMA clinical samples, we evaluated *HDAC9* nuclear expression and did not observe cytoplasmic positivity. *HDAC9* was overexpressed in our TMA SC samples and in endometrioid carcinoma samples of TCGA dataset. The conflicting results might be related to cytoplasmic or nuclear localization of *HDAC9*. In contrast to prior results from breast cancer cell-lines [13], the median *HDAC9* expression is positively associated with *ER* levels in our TMA clinical samples. *HDAC9* levels are also associated with antiestrogen resistance [13], and epigenetic silencing of *MLH1*, *PTEN* and *PgR* might be overcome by *HDAC* inhibitors [11]. The significance of *HDAC9 over*expression against resistance to hormonal treatment in endometrioid-type EC should be explored further in a larger cohort.

*DNMT3b* is involved in “de novo” methylation during early embryogenesis [32]. We demonstrated overexpression of *DNMT3b* both in TMA clinical SC-samples and TCGA dataset compared to endometrioid-type cancers. Previously, an opposing expression pattern was reported in EC cell-lines and clinical samples [17]. Low *DNMT3b* expression was also associated with better disease outcome in the EC dataset of TCGA. The latter finding is compatible with survival results from bladder and prostate cancers studies [33, 34]. *DNMT3b* is known to interact with immunologic pathways. IL6 and COX2 reduced *DNMT3b* induction and improved response to PD1 therapy in breast carcinoma [35]. There was no association between *DNMT3b* and *PD-L1* in our TMA clinical and TCGA samples. Even though the status of neither DNMT3b nor HDAC9 has association with PD-L1 levels in our cohort, their inhibitors might upregulate expression of immunostimulatory signals in tumor cells. For instance, these therapies might drive effector T cell infiltration into the tumor microenvironment by increasing cell surface expression of tumor associated antigens or by upregulating chemokines [36, 37].

In conclusion, overexpression of epigenetic markers is more common in serous-type EC. There is a positive association between BRD4 and KAT6a levels and PD-L1 expression. A combination therapy with *BRD4*, *KAT6a* and *PD-L1* blockers has a potential use in SC. MLH1 levels in association with BRD4 might have a role in predicting response to immunotherapy. It is warranted to explore further BRD4 expression against disease outcome in a larger EC cohort.

## Supporting information

S1 Appendix Details of immunohistochemical staining protocols. (DOCX)

S1 Fig ER/PgR expression in histologic EC subtypes. LEMC had the highest and CCC lowest hormonal expression levels.
(TIFF)

S2 Fig BRD4 expression against MLH1. The loss of MLH1 expression is associated with low BRD4 H-scores.
(TIFF)

S3 Fig The association of PD-L1 with KAT6a, BRD4 and PgR in TCGA data. (TIFF)

S4 Fig BRD4 and KAT6a correlation (r = 0.48) in TCGA data. (TIFF)

S1 Table Summary of clinical data. (XLSX)

S2 Table MLH1 expression in clinical samples and the association between MLH1 and PD-L1. (DOCX)
