# Supplementary material for: Expression of epigenetic pathway related genes in association with PD-L1, ER/PgR and MLH1 in endometrial carcinoma
Source: PLoS One. 2022 Feb 28;17(2):e0264014. doi: 10.1371/journal.pone.0264014 (PMC8884513; doi:10.1371/journal.pone.0264014)
Supplement: S1 Appendix — (DOCX) [file pone.0264014.s001.docx]

***HDAC9*** Immunohistochemical staining

Slides were stained using a Leica Bond RX automated system (Leica Biosytems, Buffalo Grove, IL). Slides were deparaffinized on the automated system with Dewax Solution (Leica). Heat induced antigen retrieval was performed with Epitope Retrieval Solution 2 at 10 minutes (Leica). The mouse primary HDAC9 antibody (#MA5-26729, Invitrogen, Carlsbad, CA) was used at a 1:200 concentration in Dako antibody diluent (Carpenteria, CA) and incubated for 15 min. The Leica Bond Polymer Refine Detection System was used. Polymer incubation was done for 8 min. Slides were then counterstained with Hematoxylin, dehydrated and coverslipped.

Control tissue: Endometrial adenocarcinoma.

**BRD4** Immunohistochemical staining

Slides were stained using a Leica Bond RX automated system (Leica Biosytems, Buffalo Grove, IL). Slides were deparaffinized on the automated system with Dewax Solution (Leica). Heat induced antigen retrieval was performed with Epitope Retrieval Solution 1 at 20 minutes (Leica). The rabbit primary BRD4 antibody (#ab128874, Abcam, Cambridge, MA) was used at a 1:200 concentration in Dako antibody diluent (Carpenteria, CA) and incubated for 15 min. The Leica Bond Polymer Refine Detection System was used. Polymer incubation was done for 8 min. Slides were then counterstained with Hematoxylin, dehydrated and coverslipped.

Control tissue: Colon cancer

**ER**  *(Ventana #790-4324)* Immunohistochemical staining

Slides were stained using a Ventana Discovery XT automated system (Ventana Medical Systems, Tucson, AZ). Briefly, slides were deparaffinized on the automated system with Discovery Wash solution (Ventana). Heat-induced antigen retrieval method was used in Cell Conditioning 1 (Ventana). The rabbit primary ER antibody (#790-4324, Roche, Tucson, AZ) was used at ready-to-use concentration and incubated for 16 min. The Ventana OmniMap Anti-Rabbit Secondary Antibody was used for 16 min. The detection system used was the Ventana ChromoMap kit and slides were then counterstained with Hematoxylin, dehydrated and coverslipped.

Control tissue: breast cancer

**PgR** *(Ventana #790-2223)* Immunohistochemical staining

 Slides were stained using a Ventana Discovery XT automated system (Ventana Medical Systems, Tucson, AZ). Slides were deparaffinized on the automated system with Discovery Wash solution (Ventana). Heat-induced antigen retrieval method was used in Cell Conditioning 1 (Ventana). The rabbit primary PgR antibody (790-2223, Ventana, AZ) was used at a prediluted concentration and incubated for 24 min. The Ventana OmniMap Anti-Rabbit Secondary Antibody was used for 16 min. The detection system used was the Ventana ChromoMap kit and slides were then counterstained with Hematoxylin, dehydrated and coverslipped.

Control tissue: breast cancer.

***KAT6A*** *Immunohistochemical staining*

 Slides were stained using a Ventana Discovery XT automated system (Ventana Medical Systems, Tucson, AZ). Briefly, slides were deparaffinized on the automated system with Discovery Wash solution (Ventana). Heat-induced antigen retrieval method was used in Cell Conditioning 1 (Ventana). The rabbit primary KAT6 antibody ( #PA5-66566, Invitrogen, Carlsbad, CA) was used at a 1:200 concentration in Dako antibody diluent (Carpenteria, CA) and incubated for 32 min. The Ventana OmniMap Anti-Rabbit Secondary Antibody was used for 16 min. The detection system used was the Ventana ChromoMap kit and slides were then counterstained with Hematoxylin, dehydrated and coverslipped.

Control Tissue: Colon (normal)

***DNMT3b*** *Immunohistochemical staining*

Slides were stained using a Ventana Discovery XT automated system (Ventana Medical Systems, Tucson, AZ). Briefly, slides were deparaffinized on the automated system with Discovery Wash solution (Ventana). Heat-induced antigen retrieval method was used in Cell Conditioning 1 (Ventana). The rabbit primary DNMT3b antibody (#ab227833, Abcam, Cambridge, MA) was used at a 1:100 concentration in Dako antibody diluent (Carpenteria, CA) and incubated for 60 min. The Ventana OmniMap Anti-Rabbit Secondary Antibody was used for 8 min. The detection system used was the Ventana ChromoMap kit and slides were then counterstained with Hematoxylin, dehydrated and coverslipped.

Control tissue: colon (normal).

**PD-L1** *Immunohistochemical staining*

Slides were stained using a Ventana Discovery XT automated system (Ventana Medical Systems, Tucson, AZ). Briefly, slides were deparaffinized on the automated system with Discovery Wash solution (Ventana). Heat-induced antigen retrieval method was used in RiboCC (Ventana). The rabbit primary PD-L1 antibody (#13684, Cell Signaling Technologies, Danvers, MA) was used at a 1:50 concentration (no heat) in Dako antibody diluent (Carpenteria, CA) and incubated for 2 hours. The Ventana OmniMap Anti-Rabbit Secondary Antibody was used for 16 min. The detection system used was the Ventana ChromoMap kit and slides were then counterstained with Hematoxylin.  Slides were then dehydrated and coverslipped.

Control tissue: Placenta
