# Supplementary figures and images for: Expression of epigenetic pathway related genes in association with PD-L1, ER/PgR and MLH1 in endometrial carcinoma
Source: PLoS One. 2022 Feb 28;17(2):e0264014. doi: 10.1371/journal.pone.0264014 (PMC8884513; doi:10.1371/journal.pone.0264014)

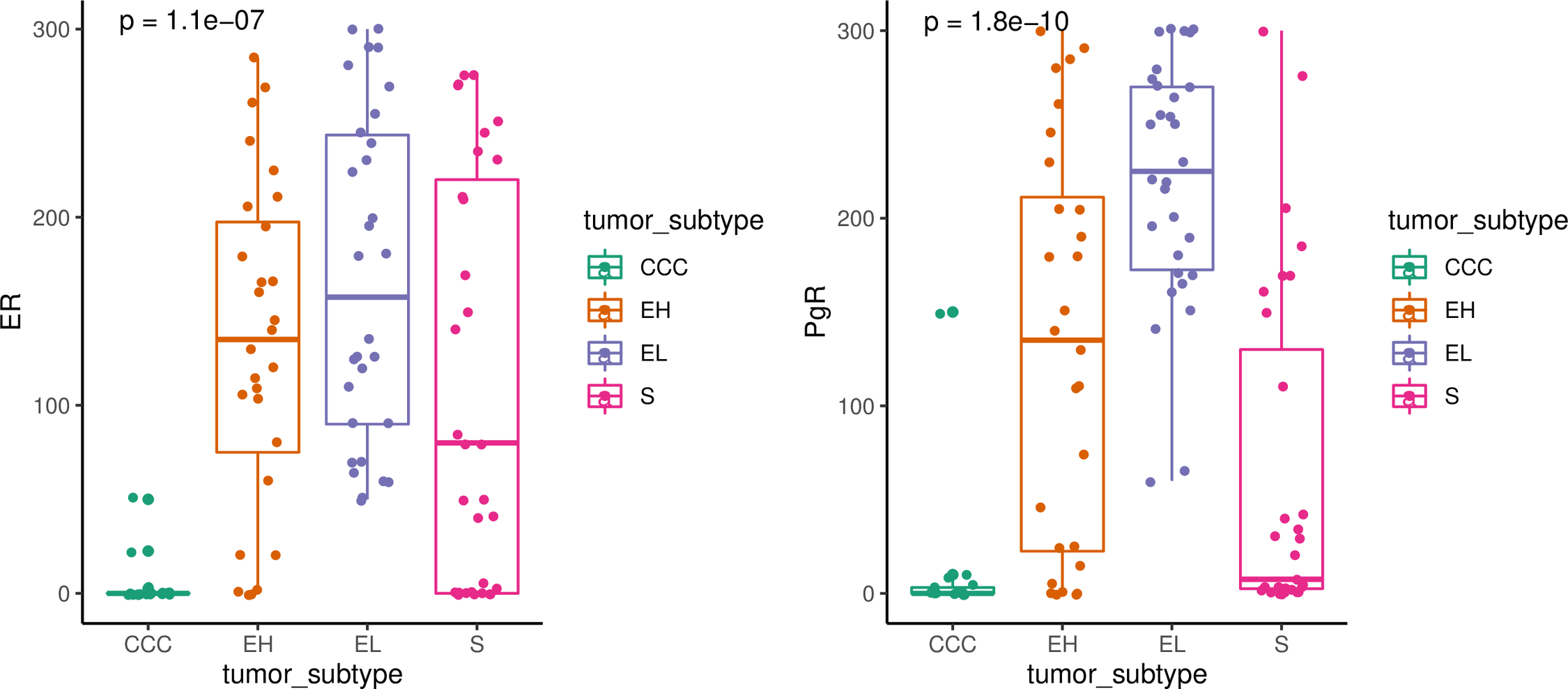

Supplement: S1 Fig — LEMC had the highest and CCC lowest hormonal expression levels. (TIFF) [file pone.0264014.s002.tiff]

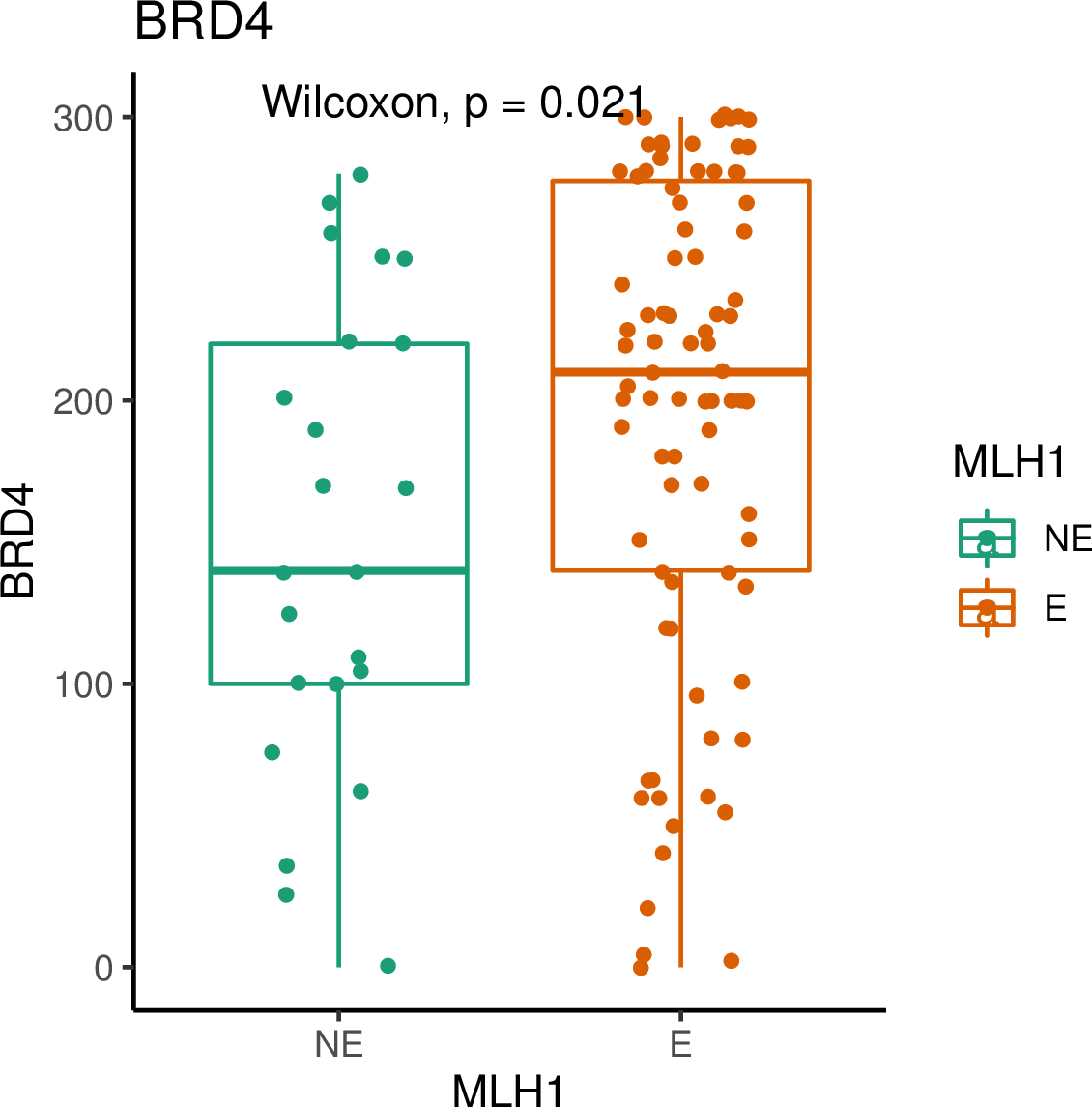

Supplement: S2 Fig — The loss of MLH1 expression is associated with low BRD4 H-scores. (TIFF) [file pone.0264014.s003.tiff]

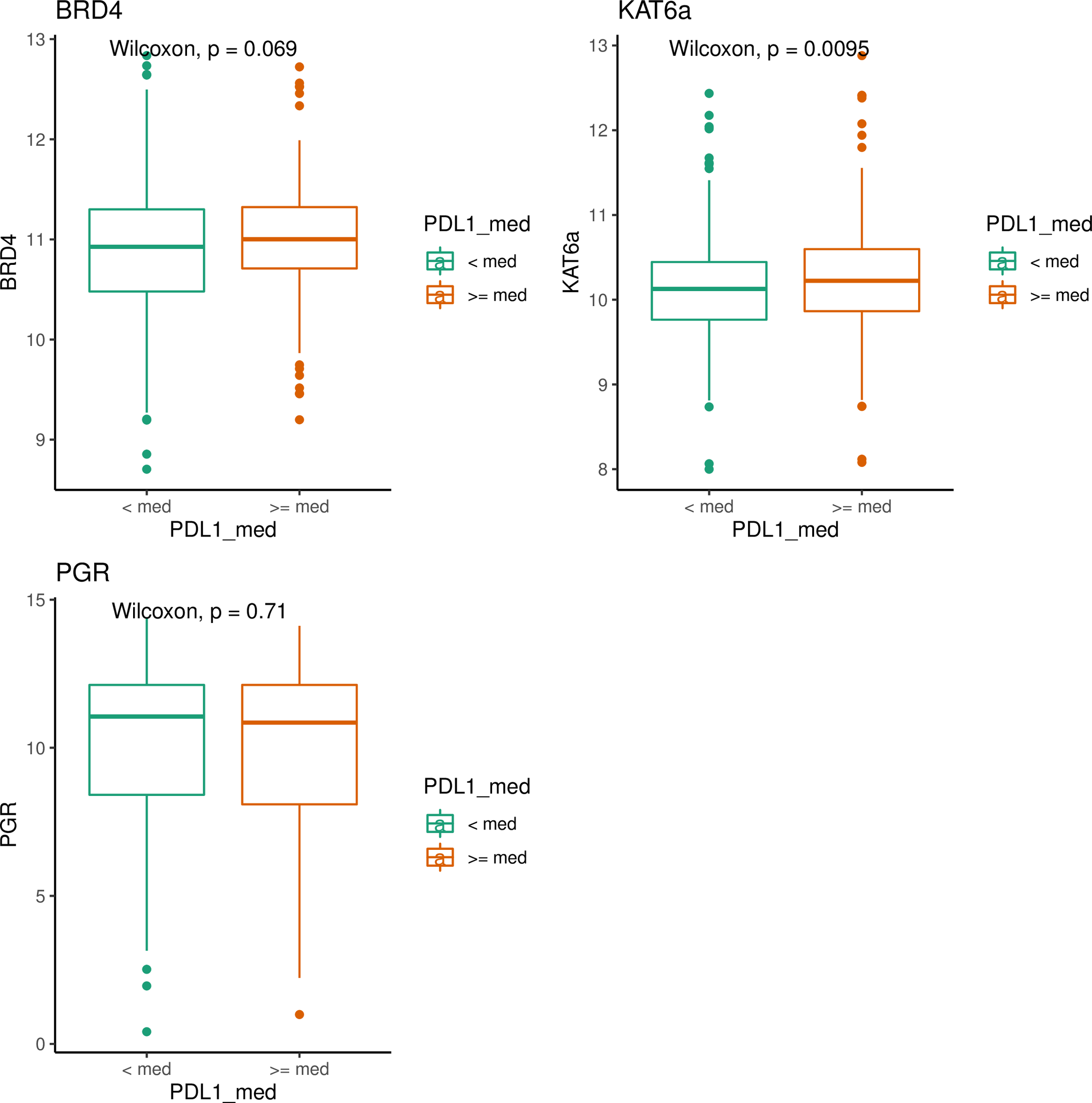

Supplement: S3 Fig — (TIFF) [file pone.0264014.s004.tiff]

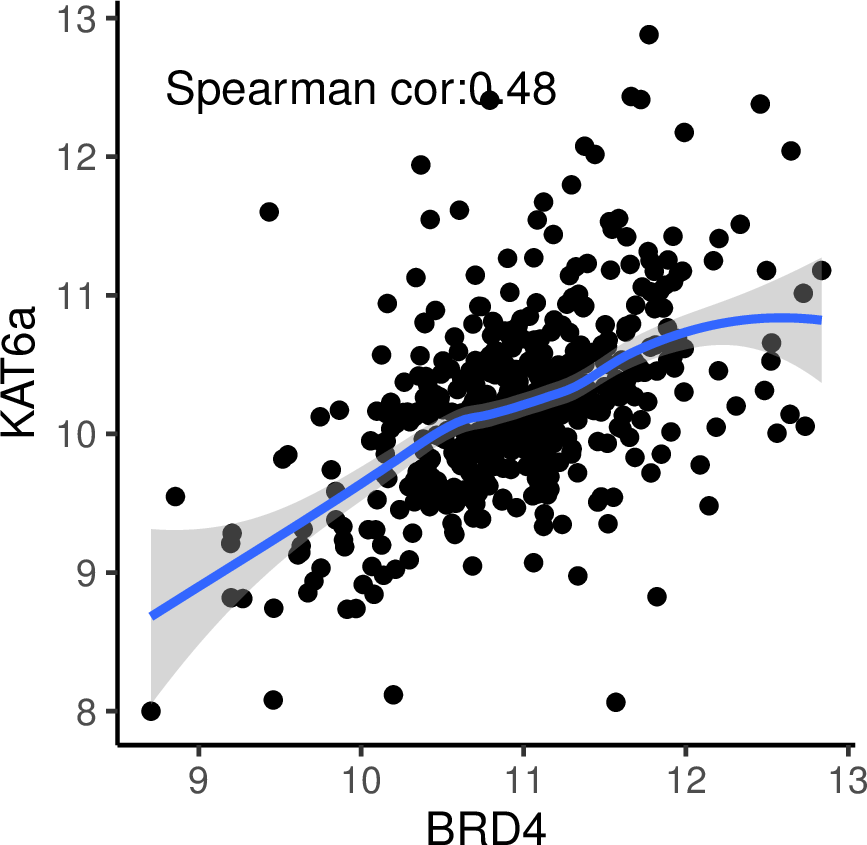

Supplement: S4 Fig — (TIFF) [file pone.0264014.s005.tiff]
