# Supplementary material for: Expression of epigenetic pathway related genes in association with PD-L1, ER/PgR and MLH1 in endometrial carcinoma
Source: PLoS One. 2022 Feb 28;17(2):e0264014. doi: 10.1371/journal.pone.0264014 (PMC8884513; doi:10.1371/journal.pone.0264014)
Supplement: S2 Table — (DOCX) [file pone.0264014.s007.docx]

|  | CCC (N = 17) | HEMC (N = 28) | LEMC (N =30) | SC (N = 31) | P |
| --- | --- | --- | --- | --- | --- |
| MLH1 |  |  |  |  | < 0.001 |
| Expressed | 16 (94.1%) | 15 (53.6%) | 21 (70.0%) | 31 (100%) |  |
| Not expressed | 1 (5.88%) | 13 (46.4%) | 9 (30.0%) | 0 (0.00%) |  |

|  | PDL1 – E (N = 44) | PDL1- NE (N = 62) | p |
| --- | --- | --- | --- |
| MLH1- Expressed | 31 (70.5%) | 52 (83.9%) | 0.158 |
| MLH1- Not expressed | 13 (29.5%) | 10 (16.1%) |  |
